# Genome-Wide Identification and Characterization of GhCOMT Gene Family during Fiber Development and Verticillium Wilt Resistance in Cotton

**DOI:** 10.3390/plants10122756

**Published:** 2021-12-14

**Authors:** Cuicui Wu, Dongyun Zuo, Shuiping Xiao, Qiaolian Wang, Hailiang Cheng, Limin Lv, Youping Zhang, Pengbo Li, Guoli Song

**Affiliations:** 1 State Key Laboratory of Cotton Biology, Institute of Cotton Research of Chinese Academy of Agricultural Sciences, Anyang 455000, China; wucuicui1982@sxau.edu.cn (C.W.); zdy041@163.com (D.Z.); shuipingxiao@163.com (S.X.); wangql1232021@163.com (Q.W.); pser2010@163.com (H.C.); llm0372@126.com (L.L.); zyp547550790@163.com (Y.Z.); 2 Cotton Research Institute, Shanxi Agricultural University, Yuncheng 044000, China; 3 Cotton Research Institute of Jiangxi Province, Jiujiang 332105, China

**Keywords:** cotton, caffeic acid *O*-methyltransferase (COMT), evolution, expression patterns, fiber development, VW

## Abstract

Caffeic acid *O*-methyltransferases (COMTs) play an essential role in lignin synthesis procession, especially in the plant’s phenylalanine metabolic pathway. The content of COMT genes in cotton and the relationship between their expression patterns have not been studied clearly in cotton. In this study, we have identified 190 COMT genes in cotton, which were classified into three groups (I, II and III), and mapped on the cotton chromosomes. In addition, we found that 135 of the 190 COMT genes result from dispersed duplication (DSD) and whole-genome duplication (WGD), indicating that DSD and WGD were the main forces driving COMT gene expansion. The Ka/Ks analysis showed that *GhCOMT43* and *GhCOMT41* evolved from *GaCOMT27* and *GrCOMT14* through positive selection. The results of qRT-PCR showed that *GhCOMT13*, *GhCOMT28*, *GhCOMT39* and *GhCOMT55* were related to lignin content during the cotton fiber development. *GhCOMT28*, *GhCOMT39*, *GhCOMT55*, *GhCOMT56* and *GhCOMT57* responded to Verticillium Wilt (VW) and maybe related to VW resistance through lignin synthesis. Conclusively, this study found that GhCOMTs were highly expressed in the secondary wall thickening stage and VW. These results provide a clue for studying the functions of GhCOMTs in the development of cotton fiber and VW resistance and could lay a foundation for breeding cotton cultivates with higher quantity and high resistance to VW.

## 1. Introduction

Cotton (*Gossypium*) is currently one of the most important economic crops globally, and it also occupies a vitally important position in economy of the China. Cotton fiber is an essential natural plant fiber with high applications in the modern textile industry [1]. Cotton fiber is developed from the single cell of ovule epidermis, and it is the most extended plant cell found so far, which is different from the general phloem fiber [2]. The process of cotton fiber development can be divided into five stages: fiber initiation stage (−3 DPA–3 DPA), rapid cell growth stage (primary wall synthesis) (2–20 DPA), transitional period (16–20 DPA), secondary wall thickening stage (16~45DPA) and dehydration maturity stage (45–50 DPA) [3,4]. The cotton fiber is mainly made of cellulose, which account for 93–95% of the dry weight, and the rest are companions of the fiber [5]. Therefore, cotton fiber is important for studying cell elongation and cell wall growth of cotton.

Lignin is a polyphenolic polymer surrounded by wood fibers, other tube bundle cells and thick-walled cell walls. It is the most prominent secondary substance in plants, and the total amount of lignin is the second-largest organic matter after cellulose [6]. Lignin can increase the mechanical strength of plant cells and tissues, help in water transport in plant tissues and improve plants’ resistance to diseases and insect pests [7,8]. In plants, there are two kinds of *O*-methyltransferases in the lignin biosynthesis pathway, namely caffeoyl coenzyme A 3-*O*-methyltransferase (CCoAOMT) and caffeoyl-CoA 3-*O*-methyltransferase (COMT) [9,10]. COMT is responsible for *O*-methylation of the C5 position of the phenol ring [10] and, therefore, plays a significant role in the lignin biosynthesis pathway [9]. For example, *AtCOMT* is a multifunctional enzyme responsible for the biosynthesis of lignin and flavonoids in plants with caffeic acid as the natural substrate [11,12]. Moreover, COMT catalyzes Melatonin production from N-acetyl 5-hydroxytryptamine [13,14]. Overexpression of *TaCOMT*s can also contribute to plants growth [15].

Few functional studies of COMT genes in some plant species [16,17,18,19,20] have revealed that COMT genes have particular impacts in different biological functions. Recently, the complete genome sequencing of two tetraploid species *G. hirsutum* [21,22,23,24,25] and two diploid cotton species *G. raimondii* [26,27] and *G. arboretum* [28] enabled analyzing the characteristics and functions of cotton COMT genes. There are two studies on the COMT genes in the *Gossypium* species [29,30]. GhCOMT1 (GenBank accession number FJ479708), GhCOMT2 (GenBank accession number FJ479709) and GhCOMT3 (GenBank accession number FJ848869) were highly expressed in roots and stems of cotton but not in leaves and cotyledons [29]. Another study showed that *O*-methyltransferase genes have multi-responses to salt stress and fiber development in *Gossypium* species [30]. However, our study is mainly based on bioinformatics analysis of *G. hirsutum*, *G. barbadense*, *G. arboretum* and *G. raimondii*, which contained the identification of COMT family genes, chromosome location, conservative domain and collinear analysis and analysis of fiber development and *Verticillium wilt* response from qRT-PCR results. Thus, this study is of great significance for the study of COMT family genes for cotton fiber development and VW.

## 2. Methods

### 2.1. Identification of COMT Genes in Cotton and Subcellular Localization Prediction of GhCOMTs in G. hirsutum

The genomic, CDS and protein sequences of *G. hirsutum* (HAU), *G. arboreum* (CRI), *G. barbadense* (ZJU) and *G. raimondii* (JGI) were downloaded from Cotton Functional Genomics Database (Cotton FGD) (https://cottonfgd.org/, accessed on 10 July 2021). Genome data of *A. thaliana* [31] were downloaded for comparative analysis of COMT genes, while the Hidden Markov Model (HMM) file of the COMT conservative domain (PF00891) was obtained from the Pfam database (http://pfam.xfam.org, accessed on 10 July 2021). Then, we used HMMER3.0 software (http://www.hmmer.org/, accessed on 10 July 2021) with an e-value of 1 × 10^−5^ as the threshold to acquire COMT protein sequences, which included PF00891. All putative COMTs were further identified by SMART (http://smart.embl-heidelberg.de/, accessed on 10 July 2021), and the genes containing a COMT domain were employed for further analysis. The subcellular location of GhCOMTs was predicted by WOLF-PSORT (https://wolfpsort.hgc.jp/, accessed on 10 July 2021) [32] and CELLO ver.2.5 (http://cello.life.nctu.edu.tw/, accessed on 10 July 2021) [33].

### 2.2. Construction of Phylogenetic Tree of COMT Family

Multi-sequence alignment of all COMT protein sequences was carried out using Clustal X [34], and phylogenetic trees were constructed using the MEGA software proximity method (version 6.0) (Neighbor-Joining, NJ) [35] with the calibration parameter Bootstrap being set to 1000. The online software Evolview (https://www.omicsclass.com/article/671, accessed on 10 July 2021) was used to modify the evolutionary tree.

### 2.3. Chromosome Location, Collinearity Analysis and Gene Duplication Analysis

Genomic sequences, CDS sequences, and GFF (general feature format) information of *G. hirsutum*, *G. arboreum* and *G. raimondii* were downloaded from Cotton Functional Genomic Database (CottonFGD) (https://cottonfgd.org/, accessed on 10 July 2021). The position of COMT members on chromosomes was analyzed and drawn by MapChart2.2 software. To determine whether the COMT gene family expanded through segmental duplication or tandem duplication events, a collinear analysis was completed with an all-to-all BLAST array (E-value of 1 × 10^−5^) in the MCScan program [36]. They were visualized using TBtools software, and the parameter for filtering genes in the small collinearity block was set to 40 [37].

### 2.4. Gene Duplication and Synonymous and Non-Synonymous Substitution Rate

Gene duplication in cotton species was executed by DupGen_finder (https://github.com/qiao-xin/DupGen_finder, accessed on 10 July 2021). Selection pressure analysis was performed by calculation of Ka (non-synonymous substitution rate) and Ks (synonymous substitution rate) values of homologous genes using KaKs_Calculator 2.0 software [38], with NG methods to calculate the Ks/Ka ratios of homologous gene pairs.

### 2.5. Gene Structure and Conservative Protein Motif

The exon–intron structure of GhCOMTs family genes was analyzed with the online Gene Structure Display Server (GSDS) [39] (http://gsds.cbi.pku.edu.cn, accessed on 10 July 2021) by inputting gene annotation GFF files. The MEME program [40] was used to analyze conserved motifs of entire GhCOMT protein sequences with the following parameters: a maximum number of ten motifs, an optimum motif width of 6 to 50, the motif number distributed in sequences was set to 0 or 1 and the remainder of the parameters was set to system defaults. The phylogenetic tree, along with gene structure, and conserved protein motifs were combined and visualized by TBtools [37].

### 2.6. Analysis of COMT Gene Promoters in the Cotton

The elements in the promoter fragments of the GhCOMT genes (1500 bp upstream region of the initiation codon “ATG” of GhCOMTs) were identified to perform the cis-acting element analysis with the online program PlantCARE (http://bioinformatics.psb.ugent.be/webtools/plantcare/html/, accessed on 10 July 2021). The promoters of GhCOMTs with specific expression pattern were referred as target sequences. Homer [41] was used to identify the specific cis-elements in the target sequences.

### 2.7. Analysis of Expression Pattern of GhCOMTs during Fiber Development

The TM-1 ovules at −3 DPA, −1 DPA, 0 DPA, 1 DPA, 3 DPA and 5 DPA and fibers at 7 DPA, 10 DPA, 15 DPA, 20 DPA and 30 DPA were collected. Three biological repeats were collected in each stage. All samples were immediately frozen in liquid nitrogen and stored at −80 °C. Total RNAs were extracted by the Total RNA Extraction Kit (R1200) (Beijing Solarbio Science & Technology Co. Ltd., Beijing, China) from all the samples for transcriptomic analysis. RNA samples were used as templates for reverse transcription with the PrimeScript RT Reagent kit (Takara, Japan) to obtain cDNA libraries. The cDNA libraries were subjected to 101-cycle paired-end sequencing on an Illumina HiSeq 4000 platform at Berry Genomics (Beijing, China). The data were normalized, and FPKM (Fragments per kilobase of transcript per million) was obtained (Table S1). The GhCOMTs with an FPKM > 1 at least in one stage of the ovule and fiber development were employed for further analysis. The genes were classified into specific and nonspecific expression by the method used in previous research [42]. The expression heatmaps were visualized by TBtools software [37].

### 2.8. RNA Extraction and Real-Time Quantitative PCR Analysis for Gene Expression

*G. hirsutum* cultivars TM-1 were grown in the Institute of Cotton Research’s experimental farm, Chinese Academy of Agricultural Sciences, Anyang, Henan, China, in 2020. The total RNA samples of TM-1 ovule and fiber were extracted with the RNAprep Pure Plant Kit by (Tiangen, Beijing, China). The RNA samples were treated with DNase1 to eliminate the genomic DNA contamination. RNA concentration and integrity were observed on Nano Drop 2000 spectrophotometer (Thermo scientific, Waltham, MA, USA) and then 1% agarose gel electrophoresis was used to detect the extraction quality of RNA. An amount of 2 μg RNA was reversely transcribed into cDNA with the PrimeScript RT Reagent kit (Takara, Japan) and stored in the refrigerator at −20 °C. Specific primers were designed according to the CDS sequence of the GhCOMT genes on the primer-blast of NCBI website. The *G. hirsutum* histone-3 gene (*GhHis3*, GenBank accession no. AF024716) was used as an internal reference [43], and the primer sequences of GhCOMT genes were shown in Additional file 1: Table S2. qRT-PCR was performed with ABI 7500 fast Real-Time PCR system (Applied Biosystems, Waltham, MA, USA). Gene expression data were obtained with three biological replicates. The total reaction system was 20 μL, including 2 × SYBR Premix Ex Taq 10 μL, 10 mmol ·L^−1^ forward primer 0.5 μL, 10 mmol·L^−1^ reverse primer 0.5 μL, cDNA template 1 μL and dd H_2_O 8 μL. PCR amplification conditions were set at 95 °C, 30 s; 95 °C, 5 s; and 60 °C for 20 s. Moreover, the relative quantitative analysis of gene expression was carried out by the 2^−^^ΔΔ Ct^ method with three independent replicates [44].

### 2.9. Fungal Culture, Infection of Plants and Disease Assessment

*Vd086*, a virulent defoliating *V. dahliae* strain, was isolated from cotton in Anyang, China, and grown on PDA medium at 25 °C for 3 days. Mycelia were collected and cultured in Czapek’s medium for 3 days at 25 °C with shaking (200 rpm).

Cotton seedlings (TM-1) with one true-leaf unfolded were inoculated by dipping the roots into *Vd086* spore suspension containing 1 × 10^7^ conidia mL^−1^ [45]. The cotton root was cut with a blade and placed in a petri dish, then poured in the 10 mL spore suspensions. The control was poured into 10 mL sterile distilled water. The plants inoculated with *V. dahliae* were cultured in a growth chamber at 25 °C under a 16 h light/8 h dark cycle. The total number of inoculated plants (cotyledons and true leaves wilting or yellowing) reached more than 10. The roots, stems, cotyledons and true leaves of diseased plant and CK (5 plants, respectively, no visible wilting or yellowing symptoms) were harvested with three biological replicates to extract the total RNA, which was used to analyze the expression level of VW.

## 3. Results

### 3.1. Identification of COMT Family Members in Cotton and Protein Physico-Chemical and Biochemical Characteristics of GhCOMTs

The COMT family genes of cotton were identified by Blastp and Pfam and renamed according to the position of the genes on the chromosome. Among them, upland cotton has 57 COMT genes members named *GhCOMT1~GhCOMT57*; *G. barbadense* has the most genes and are named *GbCOMT1~GbCOMT60*; *G. arboretum* COMT members are named as *GaCOMT1~GaCOMT35*; and *G. raimondii* COMTs are named *GrCOMT1~GrCOMT35* (Additional file 1: Table S3).

We analyzed the biochemical properties of GhCOMTs (Additional file 1: Table S3). The codin sequence lengths of these genes were between 720 bp (*GhCOMT11*) to 1158 bp (*GhCOMT50*); they coded 239 (*GhCOMT11*) to 385 (*GhCOMT50*) amino acid molecules; their relative molecular masses were between 26.81 kD (*GhCOMT11*) and 43.30 kD (*GhCOMT50*); and the isoelectric points of these proteins ranged between 4.745 (*GhCOMT8*) and 7.553 (*GhCOMT27*). The subcellularlocation prediction of GhCOMTs showed that most of the proteins were located in the cell cytoplasm. Seven proteins were in the periplasmic space. *GhCOMT16* and *GhCOMT17* were in the cytoplasm and periplasmic; *GhCOMT25*, *GhCOMT26* and *GhCOMT52* were in the cytoplasm and periplasmic space (Additional file1: Table S3).

### 3.2. Phylogenetic Analysis of COMT Genes in Cotton

**Figure 1:**
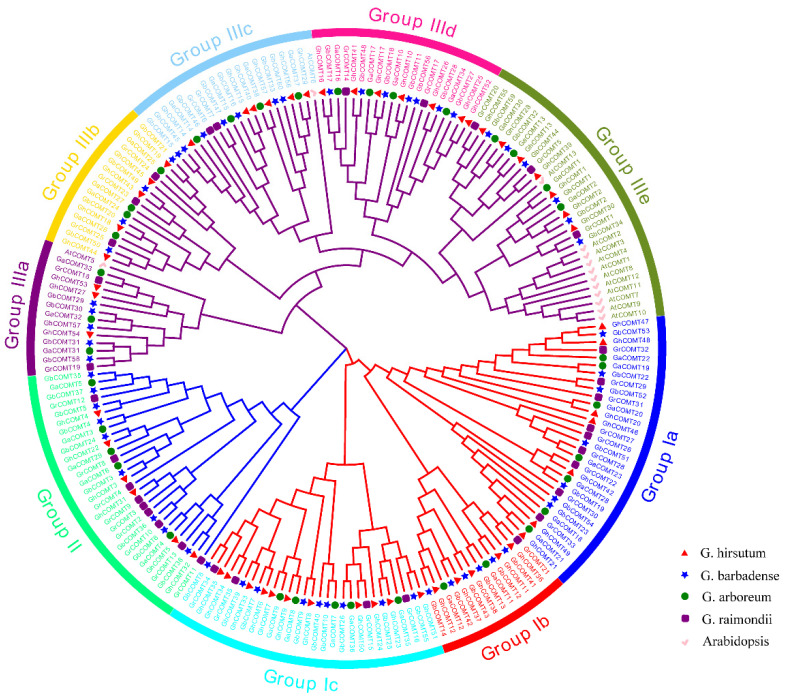
Phylogenetic analysis and subfamily classification of the COMT genes in *Arabidopsis* (AtCOMT), *G. hirsutum*(GhCOMT), *G. barbadense*(GbCOMT), *G. arboreum*(GaCOMT) and G. raimondii (GrCOMT). The phylogenetic tree was constructed with MEGA 6.0 using the neighbor-joining model with 1000 bootstrap replicates. All 190 COMTs were divided into nine subgroups, which were highlighted by different colors.

The phylogenetic tree was constructed using the amino acid sequences of all the COMT proteins between cotton and *Arabidopsis thaliana* (Figure 1). The phylogenetic tree showed that all COMT proteins were divided into three groups. The first groups were divided into three subgroups and had 73 members. The third group was divided into four subgroups and contained 100 members. All COMTs of *Arabidopsis thaliana* were distributed in the group Ⅲ, indicating that COMT genes between *Arabidopsis* and cotton differed significantly.

### 3.3. Chromosomal Distribution of Cotton COMT Genes

**Figure 2:**
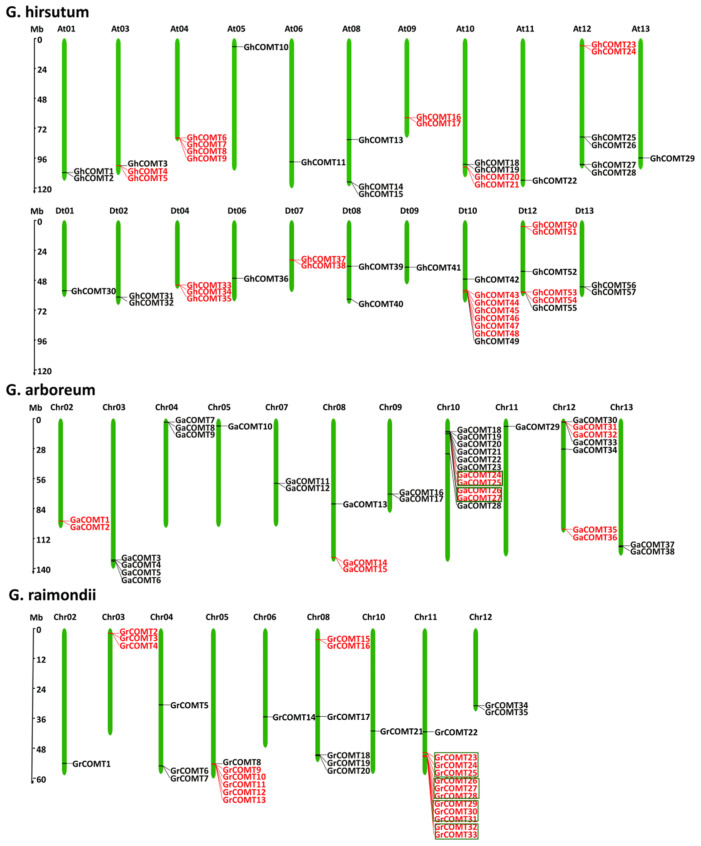
Chromosomal locations of COMT gene family in *G. hirsutum*, *G. arboreum* and *G. raimondii*. The chromosome name is above each chromosome, and the red lines on the chromosome are tandem genes. The green box separates multiple sets of tandem genes.

The analysis of chromosomal location was performed by TBtools software [37]. Fifty-six GhCOMTs, thirty-eight GaCOMTs and thirty-five GrCOMTs were positioned on their respective chromosomes (Figure 2). In *G. hirsutum* (AtDt genome), unexpectedly, there were no COMT genes in At02, At07, Dt03, Dt05 and Dt11 chromosomes (Figure 2). The distribution of genes in Dt sub-genome (28 genes) was equal with the genes in At sub-genome. The maximum number of genes in a chromosome was eight in Dt10 followed by six both in At12 and Dt12. At05, At06, At11, At13, Dt01, Dt06 and Dt09 only had one COMT gene; At01, At09, Dt02, Dt07, Dt08 and Dt13 had two COMT genes; At03, At08 and Dt04 had three COMT genes; and At04 and At10 had four COMT genes. In *G. arboretum* (A-genome) (Figure 2), 38 COMT genes were mapped in all chromosomes except chr01 and chr06. Chr10 harbored 11 COMT genes, which were the highest per chromosome, followed by chr12 and chr03 with seven and four genes, respectively. The minimum number of genes located in a chromosome was one in chr05 and chr11, respectively. In *G. raimondii* (D genome), chr11 was mapped with 12 genes followed by chr05 and chr08 with six genes. The minimum number of genes in a chromosome was one in chr2, chr6 and chr10, respectively. There was no COMT family members identified in chr01, chr07 and chr09 (Figure 2).

### 3.4. Gene Structure and Conserved Motifs Analysis of GhCOMT

**Figure 3:**
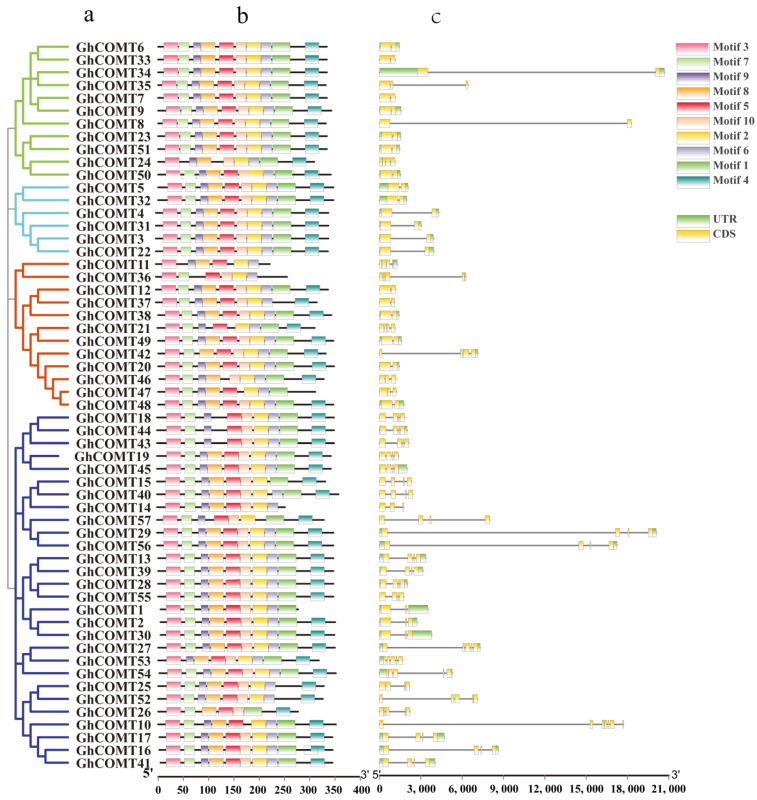
Phylogenetic relationship, conversed protein motif analysis and gene structure predictions of GhCOMT family: (**a**) a neighbor-joining phylogenetic tree. (**b**) Conversed protein motifs of the amino acid sequences of GhCOMTs. (**c**) Gene structure analysis of GhCOMT genes.

The GhCOMT gene structure helps to further investigate phylogenetic relationships (Figure 3). The GhCOMT family was divided into four groups. Within the same group, most members had similar exon and intron quantities. For example, except *GhCOMT*24 and *GhCOMT*35 which contained four and three exons, the other members in group I had two exons. In addition, 10 of the 11 members in the group had 10 motifs, and only *GhCOMT24* had eight motifs. In group II, six members contained 10 motifs and 2 exons, in which the introns lengths were different. Twelve GhCOMT genes were present in group Ⅲ, in which *GhCOMT11* and *GhCOMT36* had six motifs. In accordance with that, *GhCOMT11* and *GhCOMT36* contained four exons and five exons. Group Ⅳ had the most members—28—which contained five subgroups. *GhCOMT14*, *GhCOMT26* and *GhCOMT57* had eight, seven and seven motifs, and other members contained 9 or 10 motifs. *GhCOMT*10 consisted of seven exons, which is the maximum among the all GhCOMTs.

According to the conserved protein motifs, all members contained motif 2, 3, 5 and 6. With the exception of three members having 6–7 motifs, others all contained 8–10 motifs; thus, the GhCOMT gene family was proved to be highly conserved.

### 3.5. Collinearity Analysis of the COMT Gene Family in G. arborum, G. hirsutum and G. raimondii

**Figure 4:**
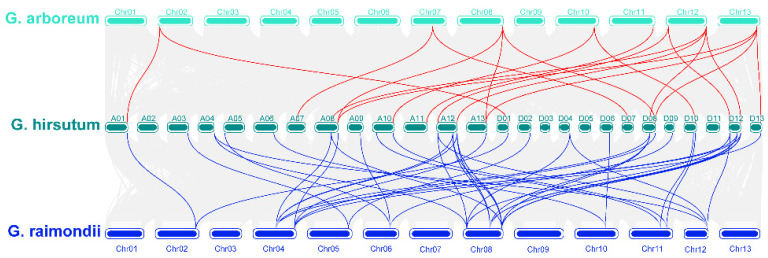
Collinearity of COMT genes between *G. hirsutum*, *G. arboretum* and *G. raimondii.* The synteny of COMTs between *G. hirsutum* and *G. arboreum* labeled with red lines and the synteny of COMTs between *G. hirsutum* and *G. raimondii* labeled with blue lines.

Gene families are forced through tandem and fragmental DNA duplication [46,47] in the whole genome evolution. We used MCScanX [36] to identify homologous gene pairs, and the result was showed in Figure 4. A total of 37 pairs of COMT genes showed collinear relationship between upland cotton and *G. arboretum*, and 32 pairs of collinear COMT genes were found between upland cotton and *G. raimondii* (Table S4). It can be seen from Figure 4 that a total of 16 GrCOMT genes had evolved into two GhCOMT genes. *GhCOMT4* and *GhCOMT31* were both derived from *GrCOMT12*, while *GhCOMT4* and *GhCOMT31* were genes on At03 and Dt02, respectively, indicating that there was a corresponding relationship between At03 and Dt02. It can also be seen from the Table S3 that 30 genes of GhCOMT were derived from 18 genes of GrCOMT, which showed a collinear relationship. Among them, *GhCOMT13*, *GhCOMT28* and *GhCOMT55* came from *GrCOMT5*. Similarly, *GhCOMT27*, *GhCOMT53* and *GhCOMT54* came from *GrCOMT18*; *GhCOMT28*, *GhCOMT39* and *GhCOMT54* came from *GrCOMT20*. It showed that one gene of *G. raimondii* could be evolved into one gene, two genes and three genes, while one gene of upland cotton may also be evolved from two genes. From the collinear distribution of COMT genes, it can be observed that one gene corresponds to multiple genes, indicating that tandem repeat events occur in the family during evolution, and this phenomenon is more common in subgroup D [21].

According to previous studies, a chromosomal region 150–200 kb in length that contained two or more genes was the evidence of a tandem duplication [48]. A total of 25 gene-pairs with segmental duplication were discovered in *G. hirsutum* (Figure 3 and Table S5). Moreover, we identified 16, 6 and 14 tandem duplicated gene-pairs in *G. hirsutum*, *G. arboretum* and *G. raimondii*, respectively (Figure 3 and Table S5). Thirty-seven and thirty-two pairs of WGD have been detected between *G. hirsutum* and *G. arboretum* and *G. hirsutum* and *G. raimondii* (Table S5). All results indicated that both segmental and tandem duplication played essential roles in the expansion of COMT family proteins, while WGDs were predominantly a duplication type between tetraploid and diploid. It can be observed that most of the tandem repeat genes occur in Dt, which is consistent with the results from 2015 where Li et al. found that Dt has a higher rate of single base mutation and non-synonymous mutation than At through sequencing cotton genome [21].

From the results of collinearity and Ka/Ks, we can observe that the COMT genes of *G. arboretum* and *G. raimondii* evolved into the COMT genes of subgroup At and subgroup Dt of upland cotton. Some genes of *G. arboretum* and *G. raimondii* would also be lost during long-term evolution. In this study, 16 COMT genes were deleted in *G. arboretum*, and 17 COMT genes were lost in *G. raimondii* (Table S4).

### 3.6. Analysis of Selection Pressure of COMT Genes in Cotton

The Ka/Ks ratio was calculated to assess selection pressure (Table S4). The ratio of Ka/Ks > 1 indicates that the gene is positively selected; thus, the non-synonymous rates are larger than the synonymous rates [46], when the ratio of Ka/Ks < 1 represents that the gene is selected negatively [46].

We analyzed Ka/Ks between *G. arboretum* and upland cotton and *G. raimondii* and upland cotton. Between *G. arboretum* and upland cotton, 37 pairs of *G. arboretum* and upland cotton had Ka/Ks values, among which the Ka/Ks value of *GrCOMT27* and *GhCOMT43* was 1.27, indicating that they evolved through positive selection. There were 11 pairs of upland cotton and *G. arboretum* with Ka/Ks values between 0.5 and 1, and 25 pairs had less than 0.5. Between *G. raimondii* and upland cotton, the rate of Ka/Ks between *GrCOMT14* and *GhCOMT41* was 1.02, indicating that *GhCOMT41* resulted from positive selection. There were seven gene pairs with Ka/Ks value between 0.5 and 1.

### 3.7. Expression Patterns of GhCOMT Genes in Fiber Development

**Figure 5:**
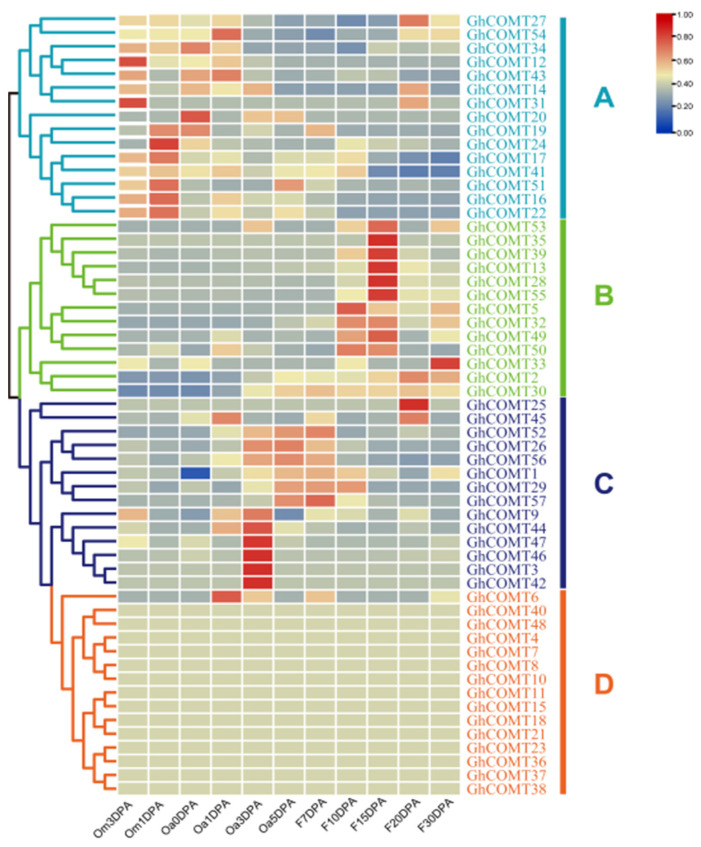
Expression profiles of GhCOMT genes in different fiber development stages. The phylogenetic relationships were displayed on the left, and the gene names were shown on the right; the fiber development stages were shown on the bottom. Scale bars at the right represented log2 (FPKM + 1). Different colours represent the different expression levels of GhCOMTs. Om3DPA: Ovule at minus 3 day post anthesis; Om1DPA: Ovule at minus 1 day post anthesis; Oa0DPA: Ovule at 0 day post anthesis; Oa1DPA: Ovule at 1 day post anthesis; Oa3DPA: Ovule at 3 day post anthesis; Oa5DPA: Ovule at 5 day post anthesis; F7DPA: Fiber at 7 day post anthesis; F10DPA: Fiber at 10 day post anthesis; F15DPA: Fiber at 15 day post anthesis; F20DPA: Fiber at 20 day post anthesis and F30DPA: Fiber at 30 day post anthesis. FRAM: fragments per kilobase of transcript per million reads.

Gene expression patterns are usually related to their functions. A transcriptome analysis of the GhCOMTs was performed using RNA-seq data for different stages of cotton ovule and fiber development (Table S1). The COMT of upland cotton can be clustered into A, B, C and D groups according to their expression in different fiber development stages (Figure 5). In group A, genes were highly expressed in the early stage of fiber development (from −3 DPA to 1 DPA). Group B played a role in the period of secondary wall thickening (10 DPA–30 DPA), and it was the period of lignin synthesis. In group C, COMT plays a role in 3 DPA–7 DPA. In group D, there were 14 COMTs that did not express at any stage of fiber development.

### 3.8. Analysis of GhCOMT Gene Promoters in the Cotton

**Figure 6:**
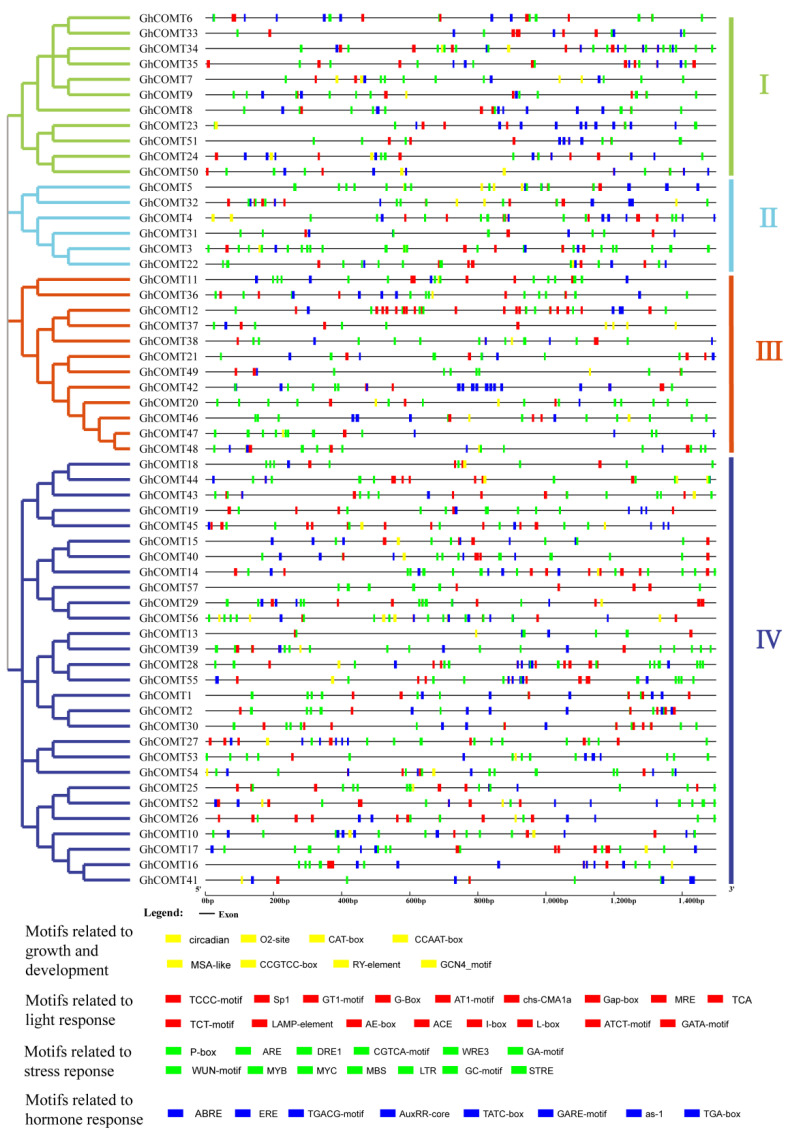
Predicted cis-elements in the promoter regions of GhCOMT genes. GhCOMTs were divided into I, Ⅱ, Ⅲ and Ⅳ according to the protein sequences. Motif names were showed below with different colors.

We analyzed the cis-acting elements on cotton COMT genes (Figure 6). The results for cotton COMT genes were similar to the blueberry COMTs [49]. According to the function, the cis-acting elements from COMT genes could be divided into four groups. Light response-related motifs constituted most cis-acting elements in cotton COMT genes and were distributed in all groups. These results showed that the COMT genes in cotton might be controlled by light. Many cis-acting elements related to plant growth and development were found in the promoter region, such as circadian, related to circadian regulatory, GCN4 motif related to endosperm development, RY-element related to seed-specific regulation and MSA-like element related to cell cycle regulation. In the motifs related to stress response, P-box was a gibberellin-responsive element that was a motif related to hormone response. MBS was involved in drought, and LTR was involved in low-temperature responsiveness.

MRE and MBS were both MYB binding sites that belonged to transcription factor binding sites. We found that AuxRR-core and TGA-box were cis-acting elements related to auxin responsiveness in the hormone response motifs. TATA-box and GARE-motif were gibberellin-responsive elements. ABRE was related to abscisic acid. Often, the type and order of cis-acting elements are similar, and the sequences have high similarity on the homologous chromosomes and high collinearity, such as *GhCOMT6* and *GhCOMT33* and *GhCOMT23* and *GhCOMT51* in group I. *GhCOMT34* and *GhCOMT35* are tandem repeat genes, and they have the similar sequence; thus, the cis-acting elements are also similar. There were many pairs of the gene that have a higher similar sequence in other groups. Similarity regulatory elements may have similar gene functions. Many GhCOMTs had LTR related to low-temperature responsiveness and GCN4 motif involved in endosperm expression. The unique regulatory elements may underlie the different functions of the genes in different groups. For example, P-box, related to gibberellin responsiveness, was mainly distributed in group I. GT1-motif, involved in light responsiveness, was mainly distributed in group II. In group III, ERE, related to hormone responsiveness, increased compared to other elements, and G-box, involved in light responsiveness, increased in group IV.

### 3.9. GhCOMT Genes Expression in Fiber Development

**Figure 7:**
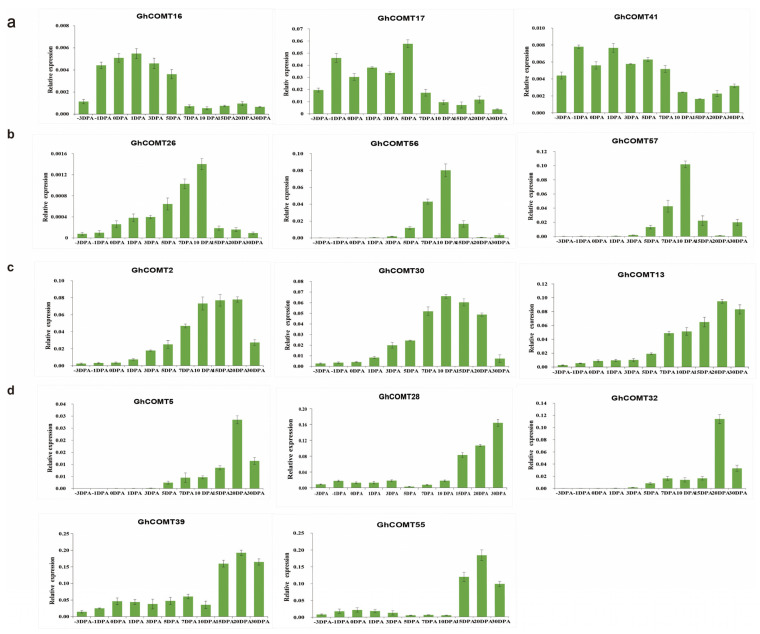
The expression patterns of 14 GhCOMTs at different development stages of cotton fibers by qRT-PCR. (**a**) Expression profiles of three GhCOMTs highly expressed in the fiber initiation and earlier stages of elongation stage. (**b**) Expression profiles of three GhCOMTs highly expressed in the fiber elongation stage. (**c**) Expression profiles of three GhCOMTs highly expressed in the fiber elongation stage and at the secondary wall synthesis stage. (**d**) Expression profiles of five GhCOMTs highly expressed at the secondary wall synthesis stage. The error bar presents standard deviations of three biological experiments. DPA: day post anthesis. *GhHis3* was used as the internal control.

According to expression in the transcriptome analysis (Figure 7), 14 GhCOMTs were selected for qRT-PCR at different fiber development stages. In Figure 5, *GhCOMT16*, *GhCOMT17* and *GhCOMT41* were in Group A, and their expressions were higher in the initial stage and earlier elongation stage of fiber development. On the other hand, *GhCOMT26*, *GhCOMT56* and *GhCOMT57* were in Group C, and their expressions were higher in the elongation stage of fiber development. In addition, *GhCOMT2*, *GhCOMT5*, *GhCOMT13*, *GhCOMT28*, *GhCOMT30*, *GhCOMT32*, *GhCOMT39* and *GhCOMT55* were in group B. The expressions of *GhCOMT5*, *GhCOMT28*, *GhCOMT32*, *GhCOMT39* and *GhCOMT55* were higher in the secondary wall thickening stage, while the expressions of *GhCOMT2*, *GhCOMT13* and *GhCOMT30* were as higher in the elongation stage and secondary wall thickening stage.

From the results of qRT-PCR, we can observe that the expression trends of *GhCOMT28*, *GhCOMT39* and *GhCOMT55* were same; the expression level was higher in 15–30 DPA and the highest is in 20 DPA. The expression of *GhCOMT13* was also highest in 20 DPA, indicating that the precursor of lignin would be synthesized the most at this time. In general, lignin synthesis is related to cellulose, but the specific relationship has not been clearly studied.

The heat map results showed that the four genes *GhCOMT13*, *GhCOMT28*, *GhCOMT39* and *GhCOM55* were clustered into one group, and their expression was highest in 15DPA (Figure 7). The relative contents of *GhCOMT13*, *GhCOMT28*, *GhCOMT39* and *GhCOMT55* are relatively high during fiber development. From the results of qRT-PCR, we can observe that the expression trend of *GhCOMT28*, *GhCOMT39* and *GhCOMT55* was the same; the expression level was higher in 15–30 DPA and the highest in 20DPA.

**Figure 8:**
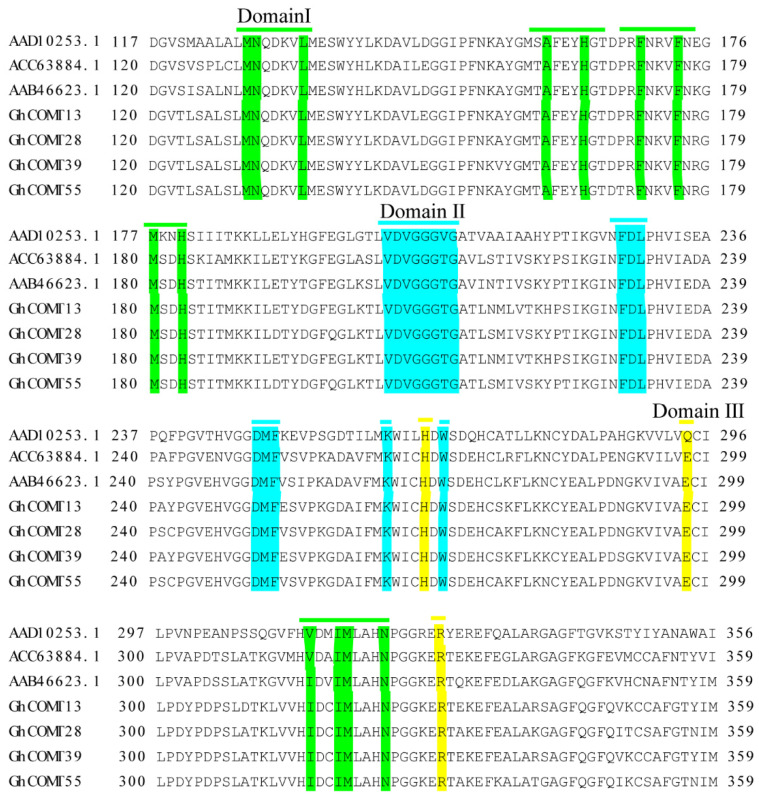
*GhCOMT13*, *GhCOMT28*, *GhCOMT39* and *GhCOM55* multiple sequence alignment was performed with other related to lignin COMT. Domain Ⅰ (Green): substrate binding site; Domain Ⅱ (Blue): SAM binding site; Domain Ⅲ (Yellow): catalytic residues.

According to the results of multiple sequences alignment (Figure 8), *GhCOMT13*, *GhCOMT28*, *GhCOMT39* and *GhCOM55* contained the same substrate binding sites with COMT that could contain catalytic caffeic acid and 5-OH coniferaldehyde [50].

### 3.10. Response of GhCOMTs in VW

**Figure 9:**
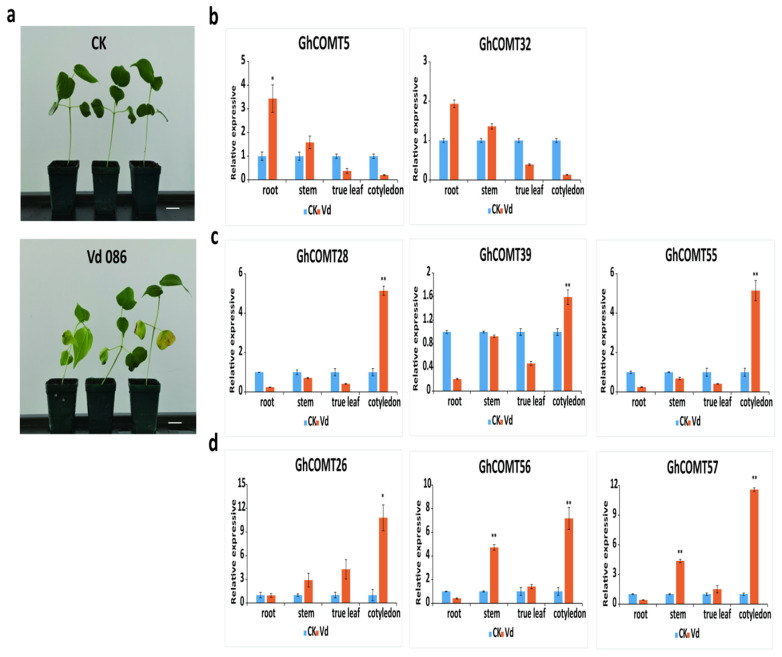
(**a**) Disease symptoms of cotton plants after 7 days inoculation with *V. dahlia*e strain Vd086. Bar = 1 cm. (**b**–**d**) shows that the expression patterns of 8 GhCOMTs at different organs with *V. dahliae* and CK(mock). The values are standardized. *GhUBQ7* (DQ116441) was used as the internal control. Each experiment was performed in three biological replicates, and the error bars represent mean ± SD; *n* = 3. * *p* < 0.05; ** *p* < 0.01.

*V. dahliae* was inoculated into TM-1. In the diseased plants and controls, we extracted RNA from roots, stems, cotyledons and true leaves and detected the expression of GhCOMT genes in these organs. The values are standardized. The results (Figure 9) showed that the expression of *GhCOMT5* and *GhCOMT32* in roots and stems of diseased plants was higher than CK, and the expression of *GhCOMT5* in roots of diseased plants was significantly higher than CK. The expressions of *GhCOMT28*, *GhCOMT39* and *GhCOMT57* in cotyledons of diseased plants were significantly higher than CK. The expressions of *GhCOMT26*, *GhCOMT56* and *GhCOMT57* in cotyledons, stems and true leaves of diseased plants were higher than CK. Among them, the expressions of *GhCOMT56* and *GhCOMT57* in cotyledons and stems of diseased plants were significantly higher than CK.

## 4. Discussion

Lignin is the basic framework of cellulose; thus, it plays an important role in woody plants, such as transporting water and enhancing resistance, etc. Lignin also plays a vital role in gramineous crops. For example, overexpression of COMT in *Arabidopsis thaliana* can promote melatonin synthesis and improve drought resistance [15]. The overexpression of *TaCOMT-3D* increased the resistance of transgenic wheat to sheath blight, stem mechanical strength and the accumulation of lignin in transgenic wheat [51]. In sorghum bmr mutants, the loss of COMT activity reduced lateral root formation and changed water restriction [20]. In addition, there are many more studies on COMT in other plants, such as tobacco [52], oatgrass [53], pine [54], rice [18], barley [19] and blueberry fruit [49]. In cotton, N-acetyltransferase 1 (*GhSNAT1*) and caffeic acid *O*-methyltransferase (GhCOMT) silencing results in a decrease in melatonin biosynthesis, which affects lignin and gossypol synthesis. It reduces resistance to *V. dahlia* [55].

Our study identified 57 GhCOMT genes in the cotton (AD)_1_ genomes, 60 GbCOMT genes in (AD)_2_ genomes, 38 GaCOMT genes in At genomes and 35 GrCOMT genes in Dt genomes. These results suggested that GhCOMT genes were lost in allotetraploid *G. hirsutum*, which was consistent with the higher rate of gene loss in allotetraploids [21,22]. According to the protein domain, 190 genes of four cotton species were divided into three groups. Group I and group II contained three subgroups, while group III had four subgroups. It can also be observed that *Arabidopsis* was herbaceous, while cotton was woody and the lignin synthesis in *Arabidopsis thaliana* and cotton should be different; thus, the distribution of COMT was also quite different. For further identifying the conservation of upland cotton, gene motifs of GhCOMTs were analyzed. Most of the genes had ten motifs, but some genes had less than ten motifs, which had different regulatory mechanisms (Figure 3). In the same family, the exons, introns and motifs had similar arrangements, which further proved the COMT classifications’ correctness. We identified these motifs, which were highly conserved in GhCOMTs. Some residues in three domains (Figure 8) (domain I: MN, L, A, H, F, F, M, H; domain II: VDVGGTG, FDL, DMF, K, W; and domain III: H, E, M, N) are related to the SAM/SAH binding site [56]. Domain I was distributed in motif 4, 5, 8 and 10 (Figure S1). Motif 2 and motif 6 of the GhCOMTs contained domain II (Figure S1). Domain III was dispersed in motif 1 and 4 (Figure S1).

Collinearity and Ka/Ks are more likely to prove tandem repetitive events. Ka/KsThe Ka/Ks of *GrCOMT*27/*GhCOMT*43 was 1.27, indicating that *GhCOMT*43 was produced by positive selection. By examining the Ka/Ks results (Table S4), it can also be seen that 21 GaCOMTsevolved into 34 GhCOMTs, and 18 GrCOMTs evolved into 30 GhCOMTs. With respect to Ka/Ks between *G. hirsutum* and *G. raimondii,* it was found that only *GhCOMT41* came from *GrCOMT14* with a Ka/Ks value of 1.01, which was the result of positive selection. The Ka/Ks value of other genes was less than 1, which were resulted from adverse selection [48]. There were two pairs of Ka/Ks values greater than one between the two diploids and upland cotton, while the Ka/Ks value of other genes was less than 0.6, indicating that the selection pressure was slight.

Go analysis showed that 50 GhCOMT genes were involved in molecular function, and there were three enrichment GO items. The molecular function includes the following aspects: (1) O-methyltransferase activity, 49 genes (98.00%); (2) methyltransferase activity, 49 genes (98.00%); and (3) protein dimerization activity, 47 genes (94.00%) (Figure S2 and Table S6). O-methyltransferase and methyltransferase activity play key roles in converting phenylalanine into lignin [9,10].

Cotton functional genomics database (https://cottonfgd.org, accessed on 10 July 2021) was used to analyze methyltransferase activity of *G. hirsutum* to determine the function and metabolic process of GhCOMTs. KEGG analysis showed that there were five enrichment KEGG pathway items. There are 25 genes matched KEGG pathway items: monolignol biosynthesis, 22 genes (88.00%); phenylpropanoid biosynthesis, 22 genes (88.00%); biosynthesis of secondary metabolites, 22 genes (88.00%); metabolic pathways, 22 genes (88.00%); and stilbenoid, diarylheptanoid and gingerol biosynthesis, 3 genes (12.00%). For a better display, we transformed the q value into an integer (−log2(q)) (Figure S3 and Table S6). Our further analysis found that 22 genes that matched monolignol biosynthesis (phenylalanine/tyrosine), phenylpropanoid biosynthesis and biosynthesis of secondary metabolites and metabolic pathways belonged to group Ⅳ, which encodes COMTs. Three genes that matched stilbenoid, diarylheptanoid and gingerol biosynthesis belonged to group Ⅲ, which encodes ROMTs (Trans-resveratrol di-O-methyltransferase). Monolignol biosynthesis consists three reference pathways: Phenylpropanoids are a group of plant secondary metabolites derived from phenylalanine. After deaminization, hydroxylation, methylation and CoA-activated, phenylalanine was converted to corresponding aldehydes and alcohols. These alcohols are called monolignols, which are the starting compounds for lignin biosynthesis. In Arabidopsis, AtCOMT is an important enzyme in lignin synthesis [11,12].

The expression profiling in eleven different fiber development periods showed that *GhCOMT13*, *GhCOMT28*, *GhCOMT39* and *GhCOMT55* had higher expression in 15DPA and may potentially function in fiber development (Figure 7). The qRT-PCR results of ovule development in −3, −1, 0, 1, 3, 5, 7, 10, 15, 20 and 30DPA showed that *GhCOMT13*, *GhCOMT28*, *GhCOMT39* and *GhCOMT55* were highly expressed at 20DPA, especially *GhCOMT28*. *GhCOMT13* and *GhCOMT39* were expressed during −3DPA to 10DPA, but *GhCOMT28* and *GhCOMT55* had lower expressions than *GhCOMT13* and *GhCOMT39*. These results showed that COMT genes had different expressions in different development periods but were highly expressed at secondary cell wall synthesis (16–21DPA). Furthermore, 18–21 DPA was critical for cell wall remodeling and synthesis of winding layer [57,58]. During fiber development, lignin content in fiber was shallow and gradually increased in the secondary wall thickening stage. The fiber mainly consisted of the ovule in the early stage, which had nearly no lignin content. The fiber cell was becoming thin and long during the rapid cell growth stage, and cellulose content gradually increased. From the secondary wall thickening stage, the fiber in the boll swells and hardens, and the water content showed a downward trend. Based on the GhCOMT fiber differential expression data from RNA-seq (Figure 7), 14 GhCOMTs were selected to detect gene expression using qRT-PCR. Previous studies have shown that lignin or lignin-like phenolics exist in cotton fibers [59,60] and contribute to the changes in the SCW and fiber strength of the transgenic lines [61,62]. The expression of COMT gene (*GhCOMT13*, *GhCOMT28*, *GhCOMT39* and *GhCOMT55*) in the periplasmic space increased and resulted in lignin accumulated in the fiber cells, which was shown by the cell wall gradually thickening [30]. Previous studies revealed that the OMT gene family was a strong growth regulator and was involved in fiber elongation and secondary cell wall synthesis stages [29]. There were three differences between this paper and Abdul et al. First of all, the reference genomes of *G. hirsutum* were different. This study used HAU genome data, and NAU genome data was analysed in Abdul’s article. Secondly, transcriptome data were different. The expessions of OMT genes were detected by using transcriptome of Chromosome Substitution Segment Lines (CSSLs) and Recombination Inbred Lines (RILs) in the previous research [29]. In this study, the heatmap of upland cotton was made by the transcriptome of standard genetic cotton cultivar TM-1, and the expression of COMT genes was detected by the cDNA of different stages of fiber development by qRT-PCR. Third, another main content was to study the relationship between COMT and VWresistance in this article, while Abdul et al. [29] studied the relationship between OMT and salt stress. The similarity between the two articles was that the sequence of *GhOMT49_At*, *GhOMT49_Dt* and *GhOMT48_At* [29] was the same as *GhCOMT13* and *GhCOMT39* and *GhCOMT28* in our study, indicating that these three genes did play an important role in fiber development. The thickening of the cell wall of cotton fiber also resulted in an increase in micronaire value [59,60]. Generally, the micronaire value of cotton was rated as Grade A in the range of 3.7–4.2, and the spinning quality was the best. Therefore, COMT gene had an essential effect on cotton fiber quality.

VW was absorbed from the root and transmitted to cotyledons and true leaves through the stem. The results of inoculation experiment of VWshowed that 6GhCOMTs responded in cotyledons, and the expression of *GhCOMT28*, *GhCOMT39*, *GhCOMT55*, *GhCOMT56* and *GhCOMT57* in cotyledons reached a very significant difference. The expression of *GhCOMT26*, *GhCOMT28*, *GhCOMT55*, *GhCOMT56* and *GhCOMT57* was almost5 times than CK, indicating that these genes responded to VW. Previous studies showed that OMT genes were induced by inoculation of *V. dahliae* and the expression patterns of phenylpropanoid changed during the inoculation of pathogens [31,63,64]. In the process of fiber development, the expression of *GhCOMT13*, *GhCOMT28*, *GhCOMT39* and *GhCOMT55* increased; thus, lignin synthesis was also increasing. At the same time, cotton plants might be infected by VW. *GhCOMT28*, *GhCOMT39*, *GhCOMT55*, *GhCOMT56* and *GhCOMT57* would respond to synthesize lignin and inhibit the expansion of VW. It had been reported that silencing caffeic acid O-methyltransferase (GhCOMT) melatonin biosynthesis genes weakened cotton resistance to VW with reduced lignin [55].

## 5. Conclusions

In this study, the COMT family in the whole genome of cotton was identified, and its gene structure, conservative structure, evolutionary relationship and collinear analysis were deeply analyzed. The results provide an essential reference value to further study the evolution and specific function of the GhCOMT gene family. Secondly, given the vital role of GhCOMT genes in the secondary wall thickening stage of *G. hirsutum* fiber development, we can speculate that GhCOMTs affect fiber strength and micronaire value and have important values for improving fiber quality and resistance to VW. Thirdly, in response to VW, it is speculated that GhCOMTs may play a role in VW resistance.

## Data Availability

The data presented in this study are available in the article and Supplementary Materials.

## Supplementary Materials

The following are available online at https://www.mdpi.com/article/10.3390/plants10122756/s1, Figure S1. The motif of GhCOMT with domains. Figure S2. The result of GO analysis. Figure S3. The result of KEGG analysis. Table S1. Primer sequences of GhCOMT genes. Table S2. Correspondence of gene name and gene ID in cotton and *Arabidopsis thaliana.* Table S3. The Ka and Ks values for homologous pairs; missing genes of *G. arboretum* and *G. raimondii.* Table S4. The FPKM value of all genes in *G. hirsutum* L.cultivar Texas Marker-1 (TM-1) during fiber development. Table S5. Genes matched GO analysis. Table S6. Genes matched KEGG pathway items.
