# Supplementary material for: Genome-Wide Identification and Characterization of GhCOMT Gene Family during Fiber Development and Verticillium Wilt Resistance in Cotton
Source: Plants (Basel). 2021 Dec 14;10(12):2756. doi: 10.3390/plants10122756 (PMC8706182; doi:10.3390/plants10122756)
Supplement: Supplementary file 1 [file plants-10-02756-s001.zip › Figure S1.pdf]

Motif

**Motif3**	**MVLKCAVELGIADIIAKAGPPATLSELAI**
**Motif7**	**NPDAPSMLDRJMRLLAHHSIF**
**Motif9**	**ERLYGLTPVSRYLVK**
**Motif8**	**EDGVSLAPLJLLMQDPVLLAPWHYLSDAV**
**Motif5**	**SPFEKAHGKDAWEYAGHBPRFNKLFNTAM**
**Motif10**	**SNHTSAIMKKCLZVYDGFZSL**
**Motif2**	**VDVGGGTGTTLSLIVSKYPQIKGINFDLP**
**Motif6**	**HVVAVAPSYPGIEHVGGDMFE**
**Motif1**	**VPKADAIFMKWVLHDWDDEECJKJLKNCYEAJPEDG**
**Motif4**	**MMAVNPGGKERTEKEWEKLARQAGFSRF**

Green: Substrate binding site;

Blue: SAM binding site;

Orange: catalytic residues
