# Supplementary material for: Genome-Wide Identification and Characterization of GhCOMT Gene Family during Fiber Development and Verticillium Wilt Resistance in Cotton
Source: Plants (Basel). 2021 Dec 14;10(12):2756. doi: 10.3390/plants10122756 (PMC8706182; doi:10.3390/plants10122756)
Supplement: Supplementary file 1 [file plants-10-02756-s001.zip › Figure S3.pdf]

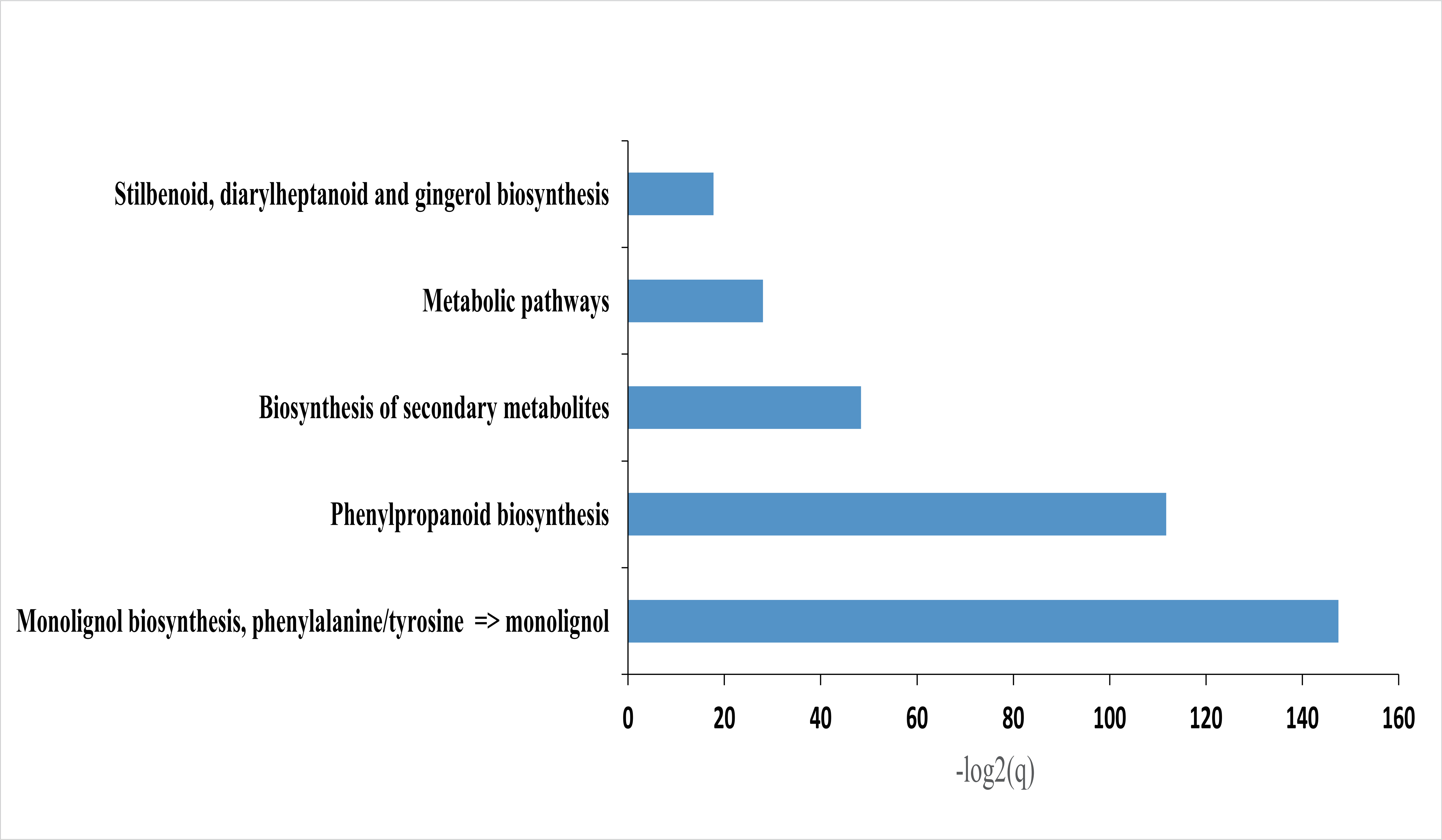

Figure S3. KEGG analysis of GhCOMTs. -log2(q): for a better display, we transformed the q value into an integer.
